# Transcriptomic and de novo proteomic analyses of organotypic entorhino-hippocampal tissue cultures reveal changes in metabolic and signaling regulators in TTX-induced synaptic plasticity

**DOI:** 10.1186/s13041-024-01153-y

**Published:** 2024-11-07

**Authors:** Maximilian Lenz, Paul Turko, Pia Kruse, Amelie Eichler, Zhuo Angel Chen, Juri Rappsilber, Imre Vida, Andreas Vlachos

**Affiliations:** 1 Department of Neuroanatomy, Institute of Anatomy and Cell Biology, Faculty of Medicine, University of Freiburg, Freiburg, Germany; 2 Hannover Medical School, Institute of Neuroanatomy and Cell Biology, Hannover, Germany; 3 Institute of Integrative Neuroanatomy and NeuroCure Cluster of Excellence, Charité-Universitätsmedizin Berlin, 10116 Berlin, Germany; 4 Chair of Bioanalytics, Technische Universität Berlin, 10623 Berlin, Germany; 5 Wellcome Centre for Cell Biology, University of Edinburgh, Edinburgh, EH9 3BF UK; 6 Si-M/“Der Simulierte Mensch”, a Science Framework of Technische Universität Berlin and Charité - Universitätsmedizin Berlin, Berlin, Germany; 7 Faculty of Medicine, Center for Basics in Neuromodulation (NeuroModulBasics) University of Freiburg,, Freiburg, Germany; 8 Center BrainLinks-BrainTools, University of Freiburg, Freiburg, Germany

**Keywords:** homeostatic synaptic plasticity, organotypic tissue culture, transcriptome, proteomics

## Abstract

Understanding the mechanisms of synaptic plasticity is crucial for elucidating how the brain adapts to internal and external stimuli. A key objective of plasticity is maintaining physiological activity states during perturbations by adjusting synaptic transmission through negative feedback mechanisms. However, identifying and characterizing novel molecular targets orchestrating synaptic plasticity remains a significant challenge. This study investigated the effects of tetrodotoxin (TTX)-induced synaptic plasticity within organotypic entorhino-hippocampal tissue cultures, offering insights into the functional, transcriptomic, and proteomic changes associated with network inhibition via voltage-gated sodium channel blockade. Our experiments demonstrate that TTX treatment induces substantial functional plasticity of excitatory synapses, as evidenced by increased miniature excitatory postsynaptic current (mEPSC) amplitudes and frequencies in both dentate granule cells and CA1 pyramidal neurons. Correlating transcriptomic and proteomic data, we identified novel targets for future research into homeostatic plasticity, including cytoglobin, SLIT-ROBO Rho GTPase Activating Protein 3, Transferrin receptor, and 3-Hydroxy-3-Methylglutaryl-CoA Synthase 1. These data provide a valuable resource for future studies aiming to understand the orchestration of homeostatic plasticity by metabolic pathways in distinct cell types of the central nervous system.

**Supplementary Information:**

The online version contains supplementary material available at 10.1186/s13041-024-01153-y.

## Introduction

Adaptations to external and internal stimuli is essential for normal brain function [1]. Neuronal plasticity can be categorized into Hebbian and homeostatic forms based on positive or negative feedback mechanisms [2]. Adjustments based on negative feedback loops are crucial for maintaining neuronal activity within a dynamic range during network perturbations [3, 4]. Numerous computational and experimental studies have highlighted their contribution to network stability and associative plasticity [5–8]. Despite extensive investigation of synaptic homeostasis across various models, the identification of novel targets orchestrating this form of plasticity remains an ongoing challenge.

Tetrodotoxin (TTX), a voltage-gated sodium channel inhibitor that suppresses network activity, has become a prevalent in vitro model for studying homeostatic synaptic plasticity [9–11]. Recent studies have identified key molecules contributing to homeostatic synaptic plasticity, such as pro-inflammatory cytokines and retinoic acid [12, 13]. Additionally, the interplay between different forms of synaptic plasticity has gained significant interest [14, 15]. Identifying novel targets involved in coordinating synaptic plasticity could further elucidate the biological significance of activity-dependent synaptic homeostasis.

In this study, we aimed to characterize transcriptomic and de novo proteomic changes associated to TTX-induced synaptic plasticity. Using whole-cell patch-clamp recordings in organotypic tissue cultures, we confirmed previous findings on TTX-induced synaptic strengthening in distinct principal neurons, specifically dentate granule cells and CA1 pyramidal neurons. We then used a combination of pulsed SILAC (Stable Isotope Labeling by Amino acids in cell culture) and homopropargylglycine (HPG) based click-chemistry to identify changes in de novo synthesized proteins following TTX treatment [16]. When combined with RNA sequencing, we found differentially expressed gene/protein pairs, particularly in metabolic pathways. These findings should act as a resource for future investigations into the mechanisms and regulatory machinery of homeostatic synaptic plasticity.

## Results

### Voltage-gated sodium channel inhibition induces functional plasticity of excitatory synapses in organotypic entorhino-hippocampal tissue cultures

**Fig. 1:**
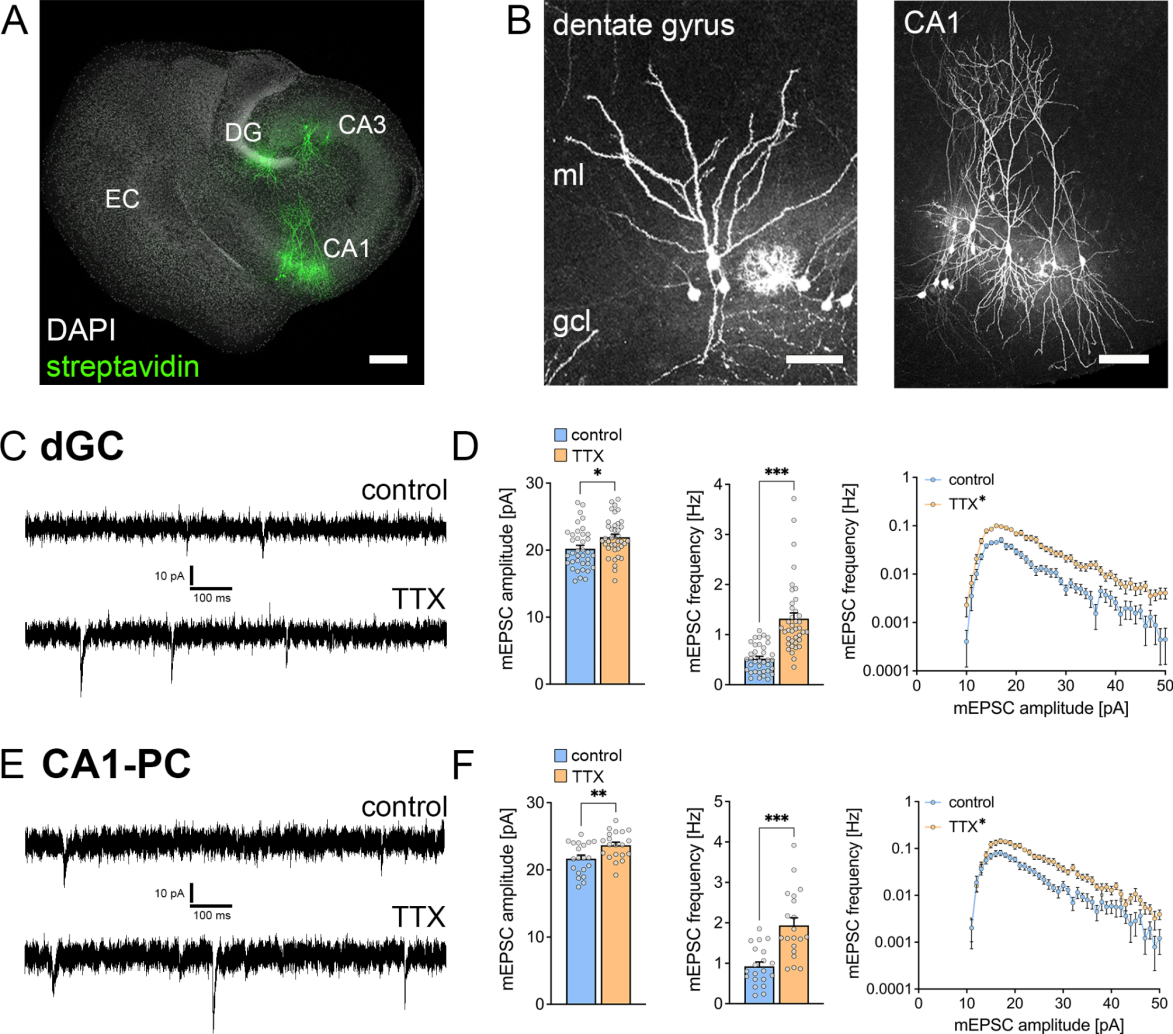
TTX treatment induces synaptic plasticity in organotypic tissue cultures. (**A**) Representative image of an organotypic entorhino-hippocampal tissue culture stained with DAPI nuclear stain and post-hoc visualization of dentate granule cells and pyramidal neurons in CA1 and CA3. EC, entorhinal cortex; DG, dentate gyrus. Scale bar, 250 μm. (**B**) Post-hoc stained dentate granule cells (left image) and CA1 pyramidal neurons (right image). gcl, granule cell layer; ml, molecular layer; Scale bar dentate gyrus, 50 μm; Scale bar CA1, 100 μm. (**C**)-(**D**) Sample traces and group data of whole-cell patch-clamp recordings of AMPA-receptor mediated miniature excitatory postsynaptic currents (mEPSCs) in dentate granule cells (dGC). Treatment with TTX (2 µM, 2 days) induces synaptic strengthening reflected by a significant increase in mEPSC amplitudes and frequencies (D; mEPSC amplitude: control, 20.2 ± 0.51 pA; TTX, 21.9 ± 0.45 pA; mEPSC frequency: control, 0.521 ± 0.045 Hz; TTX, 1.32 ± 0.115 Hz; n_control_ = 37 cells in 7 cultures, n_TTX_ = 39 cells in 7 cultures; Mann-Whitney test, RM two-way ANOVA for amplitude/frequency analysis). (**E**)-(**F**) Sample traces and group data of whole-cell patch-clamp recordings of AMPA-receptor mediated mEPSCs in CA1 pyramidal cells (CA1-PC). Treatment with TTX (2 µM, 2 days) induces synaptic strengthening reflected by a significant increase in mEPSC amplitudes and frequencies (F; mEPSC amplitude: control, 21.7 ± 0.53 pA; TTX, 23.7 ± 0.46 pA; mEPSC frequency: control, 0.925 ± 0.108 Hz; TTX, 1.94 ± 0.188 Hz; *n* = 20 cells in 4 cultures in each condition respectively; Mann-Whitney test, RM two-way ANOVA for amplitude/frequency analysis). Individual data points are indicated by gray dots. Values represent mean ± s.e.m. (*p < 0.05; **p < 0.01; ***p < 0.001)

Mouse organotypic entorhino-hippocampal tissue cultures (≥ 18 days in vitro) were treated for two days with 2 µM TTX (Fig. 1A). A significant advantage of these tissue cultures is that the cyto- and fiber architecture are preserved in vitro, enabling the assessment of different cell types within the same network (Fig. 1B). We assessed the functional effects of TTX treatment by recording α-Amino-3-hydroxy-5-methylisoxazol-4-propionsäure (AMPA) receptor mediated miniature excitatory postsynaptic currents (mEPSCs) in both dentate granule cells and CA1 pyramidal neurons (Fig. 1C, E). Consistent with previous reports, TTX treatment resulted in a significant increase in mEPSC amplitudes and frequencies in dentate granule cells (Fig. 1D). Similarly, TTX treatment led to an increase in mean mEPSC amplitude and frequency in CA1 pyramidal neurons (Fig. 1F). We concluded that TTX-mediated inhibition of voltage-gated sodium channels induces excitatory synaptic plasticity in different cell types within organotypic entorhino-hippocampal tissue cultures.

### Prolonged TTX treatment induces transcriptomic changes in organotypic tissue cultures

**Fig. 2:**
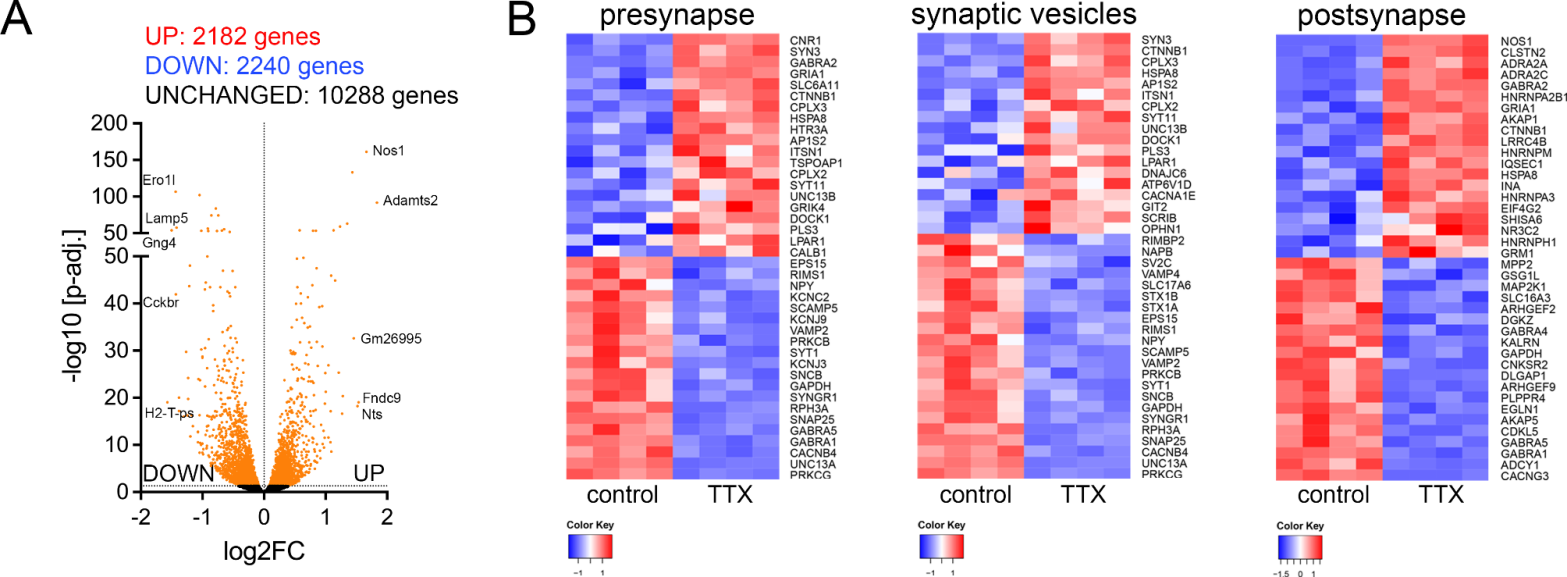
TTX treatment induces transcriptomic changes in organotypic tissue cultures. (**A**)-(**B**) RNA-Sequencing and transcriptome analysis of age- and time-matched control cultures and cultures treated with TTX (2 µM, 2 days; *n* = 4 biological replicates in each group; Table S1). (**A**) The volcano plot represents fold change and adjusted *p*-value of differentially expressed genes (indicated in orange, the Top-10 differentially expressed genes are labeled). (**B**) Heat maps of differentially expressed genes assigned to the Gene Ontology Cellular Component (GO: CC) terms “presynapse” (left panel), “synaptic vesicles” (panel in the middle), and “postsynapse” (right panel)

To further characterize the effects of TTX, we conducted a transcriptome analysis of whole tissue cultures (Fig. 2A). A substantial number of genes exhibited differential expression in response to TTX treatment compared to control cultures (UP = 2182 genes, DOWN = 2240 genes; Table S1). The gene set enrichment analysis (Figure S1, g: Profiler analysis) focused on gene ontology categories such as cellular compartment (GOCC) and biological process (GOBP), revealed differential expression of synapse and plasticity-related gene sets, including presynaptic, postsynaptic and synaptic vesicle-related genes (Fig. 2B).

### Prolonged TTX treatment is associated with changes in the synthesis of synaptic proteins

**Fig. 3:**
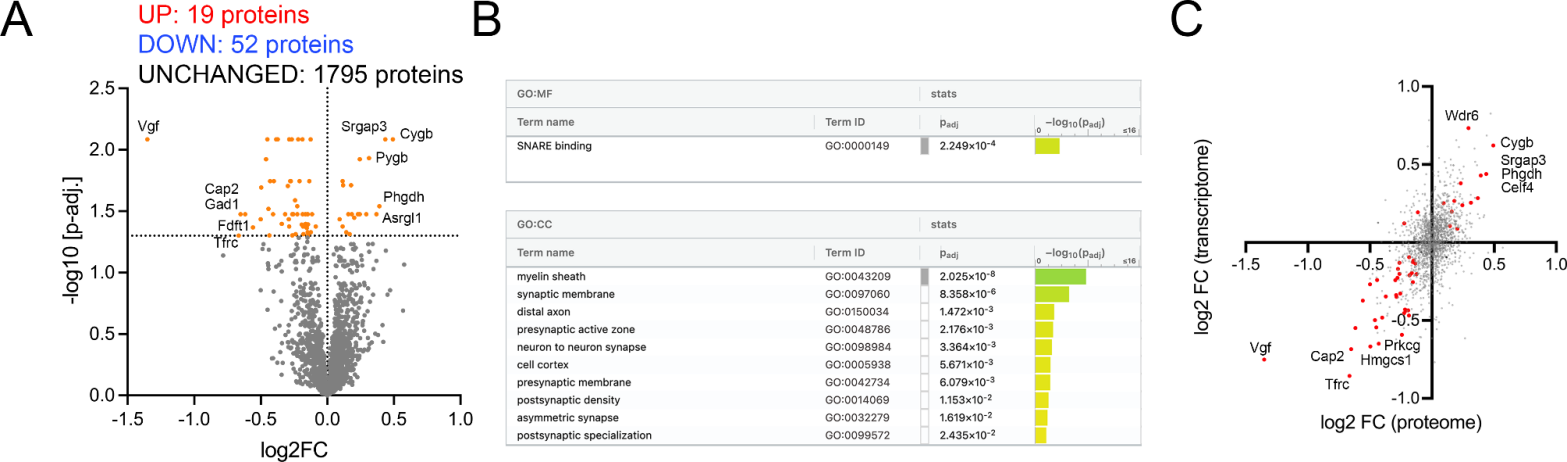
TTX treatment induces changes in de novo protein synthesis. (**A**) Volcano plot representing results of homopropargylglycine (HPG)-based stable isotope labeling with amino acids in cell culture (SILAC) analysis. Significantly up- and down-regulated proteins are indicated by colored dots. Five proteins with the highest ⎜log2FC ⎜were labeled in the volcano plot (*n* = 3 biological replicates in each group; Table S2). (**B**) g-Profiler analysis of differentially synthesized proteins upon TTX treatment. (**C**) Correlation of log2FCs from transcriptome and HPG-SILAC analyses. Significantly regulated gene/protein pairs (significance found in both transcriptome and SILAC analysis) are indicated by colored dots (Table S3)

Next, we tested for changes in de novo protein synthesis following TTX treatment. SILAC labeling was performed in combination with biorthogonal non-canonical amino acid tagging (HPG) of newly synthesized proteins in control and TTX treated tissue cultures (Fig. 3). We evaluated 1866 proteins and identified a substantial number of proteins that showed differential expression following TTX treatment (UP-regulation: 19 proteins, DOWN-regulation: 52 proteins, unchanged: 1795 proteins; Fig. 3A). In addition to synapse-related terms, such as SNARE binding (MF, GO: 0000149) or synaptic membrane (CC, GO: 0097060), g-Profiler analysis revealed a significant enrichment in the term ‘myelin sheath’ (CC, GO: 0043209; Fig. 3B). We then performed a correlation of transcriptome and proteome analyses to identify concordant matches in gene expression upon TTX treatment (Fig. 3C). From 1748 gene/protein pairs present in both transcriptome and proteome analyses, 50 gene/protein pairs were significantly regulated in both experimental settings: While 37 gene/protein pairs show a concordant down-regulation and 11 a concordant up-regulation, 2 gene/protein pairs were discordantly regulated (Table S3). In this approach, novel targets for future investigations of TTX-induced homeostatic plasticity were identified, such as cytoglobin (Cygb, UP), SLIT-ROBO Rho GTPase Activating Protein 3 (Srgap3, UP), Transferrin receptor (Tfrc, DOWN), and 3-Hydroxy-3-Methylglutaryl-CoA Synthase 1 (Hmgcs1, DOWN). We therefore regard this data set as a resource for the investigation of silencing-induced synaptic plasticity mechanisms.

## Discussion

Inhibition of voltage-gated sodium channels through TTX treatment in organotypic entorhino-hippocampal tissue cultures led to a robust functional plasticity of excitatory synapses, evidenced by increased mEPSC amplitudes and frequencies in dentate granule cells and CA1 pyramidal neurons [17, 18]. Transcriptomic analysis revealed differential expression in over 4000 genes, with significant changes in synapse and plasticity-related gene sets. Additionally, proteomic analysis using combined HPG-SILAC approaches showed TTX-related differential de novo synthesis of proteins in neural tissue. The concordance between transcriptomic and proteomic analyses suggests novel targets for future research on TTX-induced synaptic plasticity, offering a comprehensive resource for understanding silencing-induced synaptic mechanisms.

Our investigation into TTX-induced synaptic plasticity utilized organotypic entorhino-hippocampal tissue cultures [19, 20]. These cultures, once matured in vitro, exhibit a state of equilibrium where the synaptic architecture mirrors in vivo conditions [21]. Specifically, hippocampal tissue maintains functional in vivo-like connectivity, such as the perforant path targeting distal apical dendrites of hippocampal principal cells [22, 23]. Additionally, neuron-glia-interactions, essential for regulating synaptic plasticity, remain intact in these culture preparations [24, 25]. While this experimental setting offers significant advantage over primary dissociated cell cultures, it is important to note that it lacks various environmental factors that naturally influence synaptic function and plasticity within the hippocampus, such as commissural and septal/subcortical projections and intact vasculature.

Initially, TTX-induced synaptic plasticity was conceptualized as a uniform, neuron-wide negative-feedback mechanism aimed at regulating synaptic strength across all synapses of a neuron [11, 8]. However, subsequent research has substantiated the occurrence of homeostatic synaptic plasticity at more localized scales, affecting specific groups of synapses (e.g [26, 27, 18]. The significance of homeostatic synaptic plasticity, particularly in pathological conditions characterized by altered network activity and partial denervation, is increasingly recognized for restoring functional network equilibrium [28–30]. Unravelling the coordination of different forms of synaptic adjustments, such as homeostatic and Hebbian plasticity, remains an ongoing challenge [31, 15]. Recent evidence has shown that adaptations to changing activity levels exhibit multiphasic time courses, recruiting both Hebbian and homeostatic mechanisms [14]. Additionally, the synergistic actions of homeostatic plasticity in memory formation and specificity have gained acknowledgment in recent years [32].

To identify novel targets for future investigations in TTX-induced synaptic plasticity, we combined functional assessment of excitatory neurotransmission with transcriptome and proteome analyses. Using an HPG-SILAC approach, which combines non-canonical and stable isotope amino acid labeling, to label de novo synthesized proteins upon TTX treatment [33, 34]. Our results identified novel targets such as cytoglobin (Cygb), involved in nitric oxide metabolism and oxidative stress regulation; [35] or hydroxmethylglutaryl-CoA synthase (Hmgcs1), involved in mevalonate synthesis and cholesterol metabolism. Of note, previously identified factors for hippocampal synaptic plasticity, such as the BDNF-regulating neuropeptide VGF were found as well in our analysis [36, 37]. These findings suggest roles for distinct metabolic pathways and indicate differential contributions of specific cell populations: Cygb is predominantly found in interneurons, while Hmgcs1 is highly expressed in principal neurons. However, our data lack spatial resolution, which is necessary to evaluate changes in various CNS cell types, such as oligodendrocytes or microglia.

In conclusion, this study offers a valuable resource for identifying novel targets in TTX-induced synaptic plasticity research. We are confident our findings will enhance the understanding of how homeostatic plasticity is orchestrated by various metabolic pathways in distinct cell types.

## Materials and methods

### Ethics statement

Experimental procedures with animals were performed according to German animal welfare legislation and the ARRIVE guidelines, and approved by the appropriate animal welfare committee and the animal welfare officer of the Albert-Ludwigs-Universität Freiburg (University of Freiburg), Faculty of Medicine (X-17/07K, X-21/01B; preparation of organotypic tissue cultures). Mice were obtained from the Center for Experimental Models and Transgenic Services (*CEMT*, University of Freiburg). Mice were maintained in a 12 h light/dark cycle with food and water available *ad libitum*. Every effort was made to minimize distress and pain of animals.

### Preparation of tissue cultures

Organotypic entorhino-hippocampal tissue cultures were prepared from C57BL/6J mice of either sex at postnatal day 3–5 as previously described [20]. The cultivation medium contained 50% (v/v) MEM, 25% (v/v) basal medium eagle, 25% (v/v) heat-inactivated normal horse serum, 25 mM HEPES buffer solution, 0.15% (w/v) bicarbonate, 0.65% (w/v) glucose, 0.1 mg/ml streptomycin, 100 U/ml penicillin, and 2 mM glutamax. The pH was adjusted to 7.3 and the medium was replaced 3 times per week. Prior to experimental procedures, all tissue cultures were allowed to mature in a humidified atmosphere with 5% CO_2_ at 35 °C for at least 18 days.

### Pharmacology

For some experiments, tissue cultures were treated with tetrodotoxin (TTX, 2 µM, 2 d; #ab120055, Abcam) while control cultures were only treated with vehicle (water, 1 µl).

### Whole-cell patch-clamp recordings

Whole-cell patch-clamp recordings in organotypic tissue cultures were performed in a bath solution containing (in mM) 126 NaCl, 2.5 KCl, 26 NaHCO_3_, 1.25 NaH_2_PO_4_, 2 CaCl_2_, 2 MgCl_2_, and 10 glucose. In order to record miniature excitatory postsynaptic currents (mEPSCs), the solution was substituted with TTX (0.5 µM; #ab120055, Abcam), D-AP5 (10 µM; #ab120003, Abcam) and (-)-bicuculline-methiodide (10 µM; #ab120108, Abcam). Recordings were carried out at 35° under continuous oxygenation (5% CO_2_/95% O_2_) and 3–6 cells were patched per culture. Cells were visually identified using an LN-Scope (Luigs and Neumann, Germany) equipped with infrared dot-contrast and a 40× water-immersion objective (numerical aperture [NA] 0.8; Olympus). Electrophysiological signals were amplified using a Multiclamp 700B amplifier, digitized with a Digidata 1550B digitizer, and visualized with the pClamp 11 software package. Patch pipettes contained (in mM) 126 K-gluconate, 4 KCl, 10 HEPES, 4 MgATP, 0.3 Na_2_GTP, 10 PO-creatine, and 0.3% (w/v) biocytin (pH = 7.25 with KOH; 285 mOsm/kg) and had a tip resistance of 3–5 MΩ. Cells were recorded in voltage-clamp mode at a holding potential of -70 mV. Series resistance was monitored before and after recording and recordings were discarded if the resistance reached ≥ 30 MΩ.

### Post hoc staining and confocal visualization

After electrophysiological assessment, tissue cultures were fixed in 4% (w/v) paraformaldehyde (PFA; in PBS (0.1 M, pH 7.4) with 4% (w/v) sucrose) overnight. After fixation, they were washed in PBS (0.1 M, pH 7.4) and incubated with 10% (v/v) normal goat serum (NGS) in 0.5% (v/v) Triton X-100 containing PBS for 1 h, in order to reduce nonspecific staining. For post-hoc visualization of patched neurons, the tissue was incubated with Streptavidin Alexa Fluor 488 (Streptavidin A488, 1:1000; #S32354, Invitrogen) diluted in 10% (v/v) normal goat serum (NGS) in 0.1% (v/v) Triton X-100 containing PBS at 4° C overnight. After washing, the tissue was incubated with DAPI nuclear stain (1:5000 in PBS for 15 min; #62248, Thermo Scientific) in order to visualize cytoarchitecture, transferred onto glass slides and mounted with a fluorescence anti-fading mounting medium (DAKO Fluoromount; #S302380-2, Agilent). Confocal images were acquired using a Leica SP8 laser-scanning microscope equipped with a 20× multi-immersion (NA 0.75; Leica), a 40× oil-immersion (NA 1.30; Leica), and a 63× oil-immersion objective (NA 1.40; Leica). Confocal images were stored as .tif files.

### Transcriptome analysis

For experiments in organotypic tissue cultures, 5–6 cultures of the same mouse were collected and used per sample. RNA was isolated using the Monarch^®^ Total RNA Miniprep Kit (#T2010S, New England Biolabs) according to the manufacturer’s instructions. RNA quantity and quality were assessed using an Agilent RNA 6000 Pico Kit (#5067 − 1513; Agilent) with a 2100 Bioanalyzer (#G2939BA, Agilent). After RNA isolation from TTX-treated tissue cultures, library preparation and paired-end RNA sequencing (read length: 150 bp) was performed using the genome sequencer Illumina HiSeq technology in NovaSeq 6000 S4 PE150 XP sequencing mode (service provided by Eurofins). For further analysis, fastq files were provided. Data were analyzed at the Galaxy platform (usegalaxy.eu; [38]). All files contained more than 10 M high-quality reads (after mapping to the reference genome) with a phred quality of at least 30 (> 90% of total reads).

### HPG-SILAC analysis of newly synthesized proteins

For the analysis of TTX-induced changes in de novo protein synthesis, we used a homopropargylglycine (HPG) based protocol in combination with strategy for stable isotope labeling with amino acid in cell culture (SILAC). Three weeks old tissue cultures were treated with TTX (2 µM, 2 days) or vehicle-only. From 24 to 48 h, cultures were depleted from methionine, arginine and lysine through the incubation with depletion medium consisting of Neurobasal A (#041-96642 M, Gibco), B27 supplement (#17504-044, Invitrogen), penicillin/streptomycin (#15140-122, Invitrogen) and Glutamax (#35050-038, Invitrogen). The medium was supplemented with HPG (4 mM, #CLK-016-100, Jena) and stable isotope labeled amino acid with either medium or heavy weights (L-arginine – 400 µM, L-lysine – 800 µM; medium weight amino acids: Arg-6 (#CLM2265, eurisotop) and Lys-4 (#DLM2640, eurisotop); heavy weight amino acids: Arg-10 (#CNLM539, eurisotop), Lys-8 (#CDNLM-6810, eurisotop)). At the end of the treatment period, 3–6 cultures were pooled for each condition (control-medium, control-heavy, TTX-medium, TTX-heavy) and flash frozen until further processing.

To release proteins for pulldown, slices were lysed in 400 µl of Pierce RIPA buffer (#89900, Thermo Scientific) supplemented with protease inhibitor cocktail III – EDTA-free at 20 µl/ml (#539134, Merck). Control culture material fed with “HPG-medium” amino acids was mixed with TTX-treated culture material fed with “HPG-heavy” amino acids, and vice versa for the reverse condition. The mixtures were then stored on ice before being homogenized 15 times using a tissue dounce homogenizer before being repeatedly vortexed (every 5 min) over a period of 20 min. Samples were then sonicated (3 × 3-second bursts at 80% continuous power). Sonicated samples were then spun at 10,000 xg for 2 min through a cell shredder (#1011711, QIAGEN). Lysed proteins were then incubated with 200 µl of azide-agarose beads (#1038-2, Click-Chemistry Tools), in a “click-solution” containing: 0.2 mM Tris(3-hydroxypropyltriazolylmethyl)amine (THPTA, #762342, Sigma-Aldrich), 20 mM Sodium L-ascorbate (#A7631, Sigma-Aldrich) and 0.2 mM Copper(II) sulfate pentahydrate (#C8027, Sigma Aldrich) in injection grade water (AMPUWA). Azide-beads and proteins were incubated for 24 h, in the dark, on an orbital shaker (room temperature). After incubation, beads and proteins were then pelleted at 3000 x g for 3 min and washed with injection grade water. Beads and proteins were then transferred to “agarose wash buffer” (AWB; 100 mM Tris, 1% SDS, 250 mM NaCl, 5 mM EDTA, pH 8.0). To break disulfide bonds proteins were then treated for 30 min with 10 mM dithiothreitol (DTT) at 37 °C. After washing, proteins were treated for 40 min at room temperature with 400 mM Iodoacetamide (IAA), in AWB, in the dark. Following DTT and IAA treatments, beads were then repeatedly pelleted and washed (10 times with each solution) in Pierce 0.8 ml centrifuge columns (#89868, Thermo Fisher Scientific) in the following solutions: (1) agarose wash buffer, (2) 8 M Urea in 100 mM Tris, and (3) 70% acetonitrile solution (with 100 mM ammonium bicarbonate buffer; ABC). Following washing, beads were then resuspended in 10% acetonitrile in 50 mM ABC buffer. Cells were then taken for trypsin digestion and mass spectrometry analysis.

LC-MS/MS analysis was carried out using an Orbitrap Fusion Lumos Tribrid mass spectrometer (Thermo Fisher Scientific) coupled online with an Ultimate 3000 RSLCnano system (Dionex, Thermo Fisher Scientific). The sample was separated on a 50 cm EASY-Spray C18 column (Thermo Scientific) operating at 45 °C column temperature. Mobile phase A consisted of 0.1% (v/v) formic acid and mobile phase B of 80% v/v acetonitrile with 0.1% v/v formic acid. Samples for analysis were resuspended in 0.1% v/v formic acid, 1.6% v/v acetonitrile. Peptides were loaded and separated at a flow rate of 300 nl min^− 1^. Peptides were separated using a gradient with linear increases from 2% mobile phase B to 35% over 95 min then to 45% over 15 min, followed by an increase to 55% in 3 min and then a steep increase to 95% mobile phase B in 2 min.

Eluted peptides were ionized by an EASY-Spray source (Thermo Scientific) and introduced directly into the mass spectrometer. The MS data were acquired in data-dependent mode with a 3 s cycle time. For every cycle, the full scan mass spectrum was recorded in the Orbitrap at a resolution of 120 K. Ions with a precursor charge state between 2 + and 7 + were isolated and fragmented employing higher-energy collisional dissociation (HCD) with a normalized collision energy of 30% applied. The fragmentation spectra were then recorded in the ion trap with the scan rate set to “Normal”. Dynamic exclusion was enabled with a single repeat count and a 60 s exclusion duration.

### Quantification and statistics

Electrophysiological data were assessed using the pClamp 11 software package (Axon Instruments). mEPSC properties were analyzed with an automated templated-based search tool for event detection. The template was previously created in the Clampfit11 software based on manually identified and selected EPSCs. The template match threshold was set at 2.5 and the time period for the allowed duration of a single event starting from the baseline was defined. Both parameters were not changed within the different recordings or groups.

For transcriptome analysis, sequencing data were uploaded to the Galaxy web platform (public server: usegalaxy.eu), and the analysis was performed based on the RNA-seq data analysis tutorial [39]. In short, the CUTADAPT tool was used to remove adapter sequencing, low quality and short reads. Remaining reads were subsequently mapped using the RNA STAR tool with the mm10 (Mus musculus) reference genome. The evidence-based annotation of the mouse genome (GRCm38), version M25 (Ensembl 100), served as a gene model (GENCODE). An unstranded FEATURECOUNT analysis of the RNA STAR output was performed for an initial assessment of gene expression. Only samples that contained > 60% uniquely mapping reads (feature: “exon”) were considered for further analysis. Statistical evaluation was performed using DESeq2 with “treatment” as the primary factor affecting gene expression. Genes with mean reads (base mean) < 150 were excluded from further analysis. Genes with an adjusted *p*-value of < 0.05 were considered as differentially expressed. Heatmaps were generated based on the z-scores of the normalized count tables. The g: Profiler (version e107_eg54_p17_bf42210) with g: SCS multiple testing correction method (significance threshold of 0.05 [40]) was used for functional enrichment analysis of both transcriptomic and proteomic data.

Protein quantification was performed using MaxQuant (v1.6.5.0) and output data manipulated using Perseus (1.6.2.3; [41–43]. The Uniprot mouse proteome SP_UP000000589 was used as a reference database (downloaded 26/03/2021). MaxQuant search parameters were set as previously described [44], including cysteine (C) carbamidomethyl, as fixed modification, together with methionine (M) oxidation, protein N-terminal acetylation and asparagine (N) or glutamine (Q) deamination, as variable modifications. Lys0 and Arg0, Lys4 and Arg6, or Lys8 and Arg10 were set as multiplicity labels. First search and main search peptide tolerances were left as 20 and 4.5 ppm, respectively. False discovery rate was 1% for both peptide spectrum match and protein levels. Minimum peptide count was 2. Before further analysis, predicted contaminants, reverse database hits and peptides only identified by modification were excluded. Proteins with a q-value < 0.05 (FDR < 5%) were considered to be differentially expressed.

Data were statistically analyzed using GraphPad Prism 9 (GraphPad Software, USA). All values represent mean ± standard error of the mean (s.e.m.). We used a non-parametric Mann-Whitney test for comparison of two experimental groups in electrophysiological experiments. *P*-values < 0.05 were considered statistically significant (*p < 0.05, **p < 0.01, ***p < 0.001); results without statistical significance were indicated as ‘ns’. N-numbers are provided in the figure legends.

### Digital illustrations

Confocal images were stored as .tif files and image brightness and contrast were adjusted. Figures were prepared using the ImageJ software package (https://imagej.nih.gov/ij/) and Photoshop graphics software (Adobe, San Jose, CA, USA).

## Electronic Supplementary Material

Below is the link to the electronic supplementary material.

Supplementary Material 1

Supplementary Material 2

Supplementary Material 3

Supplementary Material 4

## Data Availability

Sequencing data have been deposited in the Gene Expression Omnibus (GEO) repository (accession number: GSE244095; GSM7806574-7806581). The mass spectrometry proteomics data have been deposited to the ProteomeXchange Consortium via the PRIDE partner repository [45] with the dataset identifier PXD056794. Original data are available upon reasonable request from the corresponding authors.
