# Supplementary material for: Transcriptomic and de novo proteomic analyses of organotypic entorhino-hippocampal tissue cultures reveal changes in metabolic and signaling regulators in TTX-induced synaptic plasticity
Source: Mol Brain. 2024 Nov 7;17:78. doi: 10.1186/s13041-024-01153-y (PMC11542228; doi:10.1186/s13041-024-01153-y)
Supplement: Supplementary file 1 — Supplementary Material 1 [file 13041_2024_1153_MOESM1_ESM.pdf]

***Supplementary Material*:**

Transcriptomic and de novo proteomic analyses of organotypic entorhino-hippocampal tissue cultures reveal changes in metabolic and signaling regulators in TTX-induced synaptic plasticity

Maximilian Lenz, Paul Turko, Pia Kruse, Amelie Eichler, Zhuo Angel Chen, Juri Rappsilber, Imre Vida, Andreas Vlachos

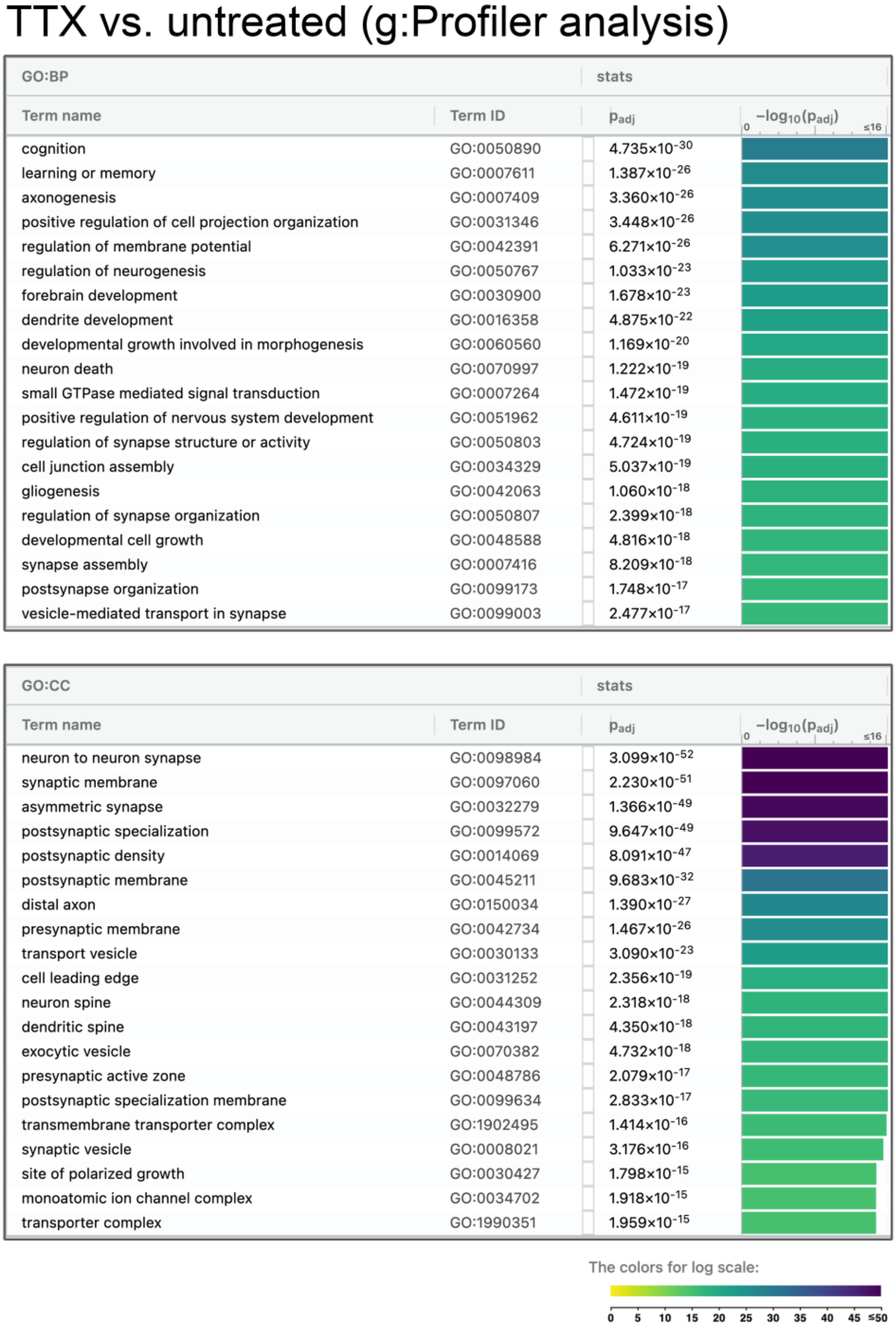

TTX vs. untreated (g:Profiler analysis)

GO:BP		stats	
Term name	Term ID	p_{adj}	$-\log_{10}(p_{adj})$
cognition	GO:0050890	4.735×10^{-30}	
learning or memory	GO:0007611	1.387×10^{-26}	
axonogenesis	GO:0007409	3.360×10^{-26}	
positive regulation of cell projection organization	GO:0031346	3.448×10^{-26}	
regulation of membrane potential	GO:0042391	6.271×10^{-26}	
regulation of neurogenesis	GO:0050767	1.033×10^{-23}	
forebrain development	GO:0030900	1.678×10^{-23}	
dendrite development	GO:0016358	4.875×10^{-22}	
developmental growth involved in morphogenesis	GO:0060560	1.169×10^{-20}	
neuron death	GO:0070997	1.222×10^{-19}	
small GTPase mediated signal transduction	GO:0007264	1.472×10^{-19}	
positive regulation of nervous system development	GO:0051962	4.611×10^{-19}	
regulation of synapse structure or activity	GO:0050803	4.724×10^{-19}	
cell junction assembly	GO:0034329	5.037×10^{-19}	
gliogenesis	GO:0042063	1.060×10^{-18}	
regulation of synapse organization	GO:0050807	2.399×10^{-18}	
developmental cell growth	GO:0048588	4.816×10^{-18}	
synapse assembly	GO:0007416	8.209×10^{-18}	
postsynapse organization	GO:0099173	1.748×10^{-17}	
vesicle-mediated transport in synapse	GO:0099003	2.477×10^{-17}	

GO:CC		stats	
Term name	Term ID	p_{adj}	$-\log_{10}(p_{adj})$
neuron to neuron synapse	GO:0098984	3.099×10^{-52}	
synaptic membrane	GO:0097060	2.230×10^{-51}	
asymmetric synapse	GO:0032279	1.366×10^{-49}	
postsynaptic specialization	GO:0099572	9.647×10^{-49}	
postsynaptic density	GO:0014069	8.091×10^{-47}	
postsynaptic membrane	GO:0045211	9.683×10^{-32}	
distal axon	GO:0150034	1.390×10^{-27}	
presynaptic membrane	GO:0042734	1.467×10^{-26}	
transport vesicle	GO:0030133	3.090×10^{-23}	
cell leading edge	GO:0031252	2.356×10^{-19}	
neuron spine	GO:0044309	2.318×10^{-18}	
dendritic spine	GO:0043197	4.350×10^{-18}	
exocytic vesicle	GO:0070382	4.732×10^{-18}	
presynaptic active zone	GO:0048786	2.079×10^{-17}	
postsynaptic specialization membrane	GO:0099634	2.833×10^{-17}	
transmembrane transporter complex	GO:1902495	1.414×10^{-16}	
synaptic vesicle	GO:0008021	3.176×10^{-16}	
site of polarized growth	GO:0030427	1.798×10^{-15}	
monoatomic ion channel complex	GO:0034702	1.918×10^{-15}	
transporter complex	GO:1990351	1.959×10^{-15}	

Figure S1: Gene-Ontology regulated terms in the RNA-sequencing of control cultures and TTX-treated cultures.

Top-10 Gene-Ontology Cellular Component (GO:CC) and Gene-Ontology Biological Process (GO:BP) regulated terms in organotypic entorhino-hippocampal tissue cultures. The color code represents the -log10(p-adj) of the differentially expressed genes.

Table S1: DeSeq2 tabular results from transcriptome analysis (n=4 biological replicates).

Table S2: HPG-SILAC analysis tabular results (n=3 biological replicates).

Table S3: Significantly regulated gene/protein pairs from transcriptome-proteome correlations.
